# Monoamines differentially modulate neuropeptide release from distinct sites within a single neuron pair

**DOI:** 10.1371/journal.pone.0196954

**Published:** 2018-05-03

**Authors:** Tobias Clark, Vera Hapiak, Mitchell Oakes, Holly Mills, Richard Komuniecki

**Affiliations:** Department of Biological Sciences, University of Toledo, Toledo, Ohio, United States of America; Biocenter, Universität Würzburg, GERMANY

## Abstract

Monoamines and neuropeptides often modulate the same behavior, but monoaminergic-peptidergic crosstalk remains poorly understood. In *Caenorhabditis elegans*, the adrenergic-like ligands, tyramine (TA) and octopamine (OA) require distinct subsets of neuropeptides in the two ASI sensory neurons to inhibit nociception. TA selectively increases the release of ASI neuropeptides encoded by *nlp-14* or *nlp-18* from either synaptic/perisynaptic regions of ASI axons or the ASI soma, respectively, and OA selectively increases the release of ASI neuropeptides encoded by *nlp-9* asymmetrically, from only the synaptic/perisynaptic region of the right ASI axon. The predicted amino acid preprosequences of genes encoding either TA- or OA-dependent neuropeptides differed markedly. However, these distinct preprosequences were not sufficient to confer monoamine-specificity and additional N-terminal peptide-encoding sequence was required. Collectively, our results demonstrate that TA and OA specifically and differentially modulate the release of distinct subsets of neuropeptides from different subcellular sites within the ASIs, highlighting the complexity of monoaminergic/peptidergic modulation, even in animals with a relatively simple nervous system.

## Introduction

Monoamines and neuropeptides interact extensively to modulate behavior in both vertebrates and invertebrates, and the disruption of monoaminergic/peptidergic crosstalk has been implicated in a range of neurological disorders, including anxiety, depression and drug abuse [1–3]. However, given the complexity of the mammalian central nervous system, the specifics of these interactions are often difficult to analyze. In contrast, *Caenorhabditis elegans* contains only 302 neurons, but still exhibits complex behaviors that are extensively modulated by both monoamines and neuropeptides, with many similarities to mammalian systems. For example, antidepressants, such as Prozac, opiates and cannabinoids all work through orthologous pathways in both systems, and the noradrenergic inhibition of pain/aversive responses is also similar, with α_2_-like adrenergic receptors inhibiting primary nociceptors and α_1_-like adrenergic receptors stimulating the release of an array of inhibitory neuropeptides [4–7]. In fact, monoamines and neuropeptides interact to modulate most, if not all, key behaviors in *C*. *elegans*. For example, aversive responses mediated by the two ASH sensory neurons are extensively modulated by monoamines and neuropeptides, and require differential monoamine-dependent peptidergic signaling from the two ASI sensory neurons. Serotonin (5-HT) stimulates the initiation of aversive responses to 1-octanol through a pathway requiring three distinct 5-HT receptors and neuropeptides encoded by *nlp-3* and *nlp-24* [5, 8, 9]. In contrast, the adrenergic-like ligands, tyramine (TA) or octopamine (OA) antagonize this 5-HT stimulation, inhibiting 5-HT-stimulated aversive responses [10, 11]. TA and OA also inhibit the initiation of aversive responses to higher repellant concentrations, but through a different subset of receptors [6, 10]. TA or OA inhibition also requires the expression of a wide range of neuropeptide-encoding genes. For example, the ASIs express Gα_q_-coupled, TA and OA receptors and require different subsets of ASI neuropeptide-encoding genes to modulate aversive responses, i.e. ASI *nlp-1*, *-14* and *-18* are required for the TA inhibition of aversive responses to low levels of repellant and *nlp-6*, *-7*, and *-9* for OA inhibition to higher levels [6, 11]. In addition, inhibitory crosstalk between the ASHs and ASIs modulates nociception and avoidance via additional serotonergic, octopaminergic and peptidergic signaling [12]. Monoaminergic and peptidergic signaling also interact to modulate feeding, nutritional status, mating, egg-laying and locomotion, as well as a host of additional behaviors [13–19]. Crosstalk among the signaling systems is extensive and complex, with monoamines and neuropeptides modulating the activity of individual neurons, entire circuits and, as described in the present study, interacting directly, with monoamines differentially regulating the release of an array of modulatory neuropeptides from somatic and synaptic sites within a single pair of neurons.

The present study was designed to define the roles of these modulatory monoamines on ASI neuropeptide release and demonstrates that individual monoamines selectively stimulate the release of distinct subsets of ASI neuropeptides from unique sites within the ASIs, with the TA-dependent neuropeptides encoded by *nlp-14* and *nlp-18* released at synaptic/perisynaptic sites or the ASI soma, respectively, and OA-dependent neuropeptides encoded by *nlp-9* released asymmetrically only from synaptic/perisynaptic sites on the right ASI axon. Together, these studies demonstrate that TA and OA differently modulate the release of distinct subsets of neuropeptides from specific sites within the ASIs and highlight the complexity of monoaminergic-peptidergic modulation.

## Results

### Distinct subsets of ASI neuropeptides are essential for the TA- and OA-dependent inhibition of aversive responses to 1-octanol

**Fig 1:**
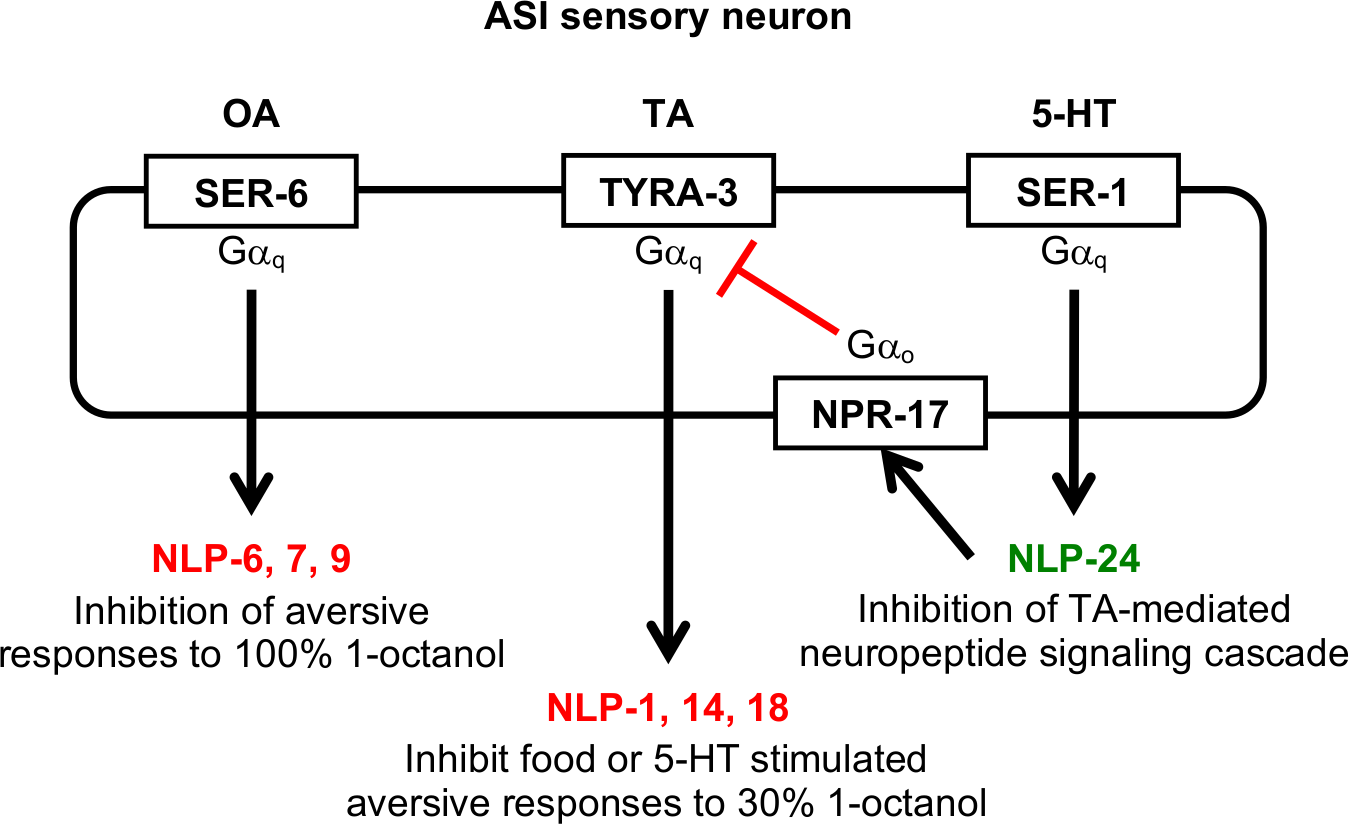
The two ASI sensory neurons integrate both monoaminergic and peptidergic signaling to modulate nociceptive behavior. The two ASI sensory neurons express Gα_q_-coupled TA, OA and 5-HT receptors that differentially modulate neuropeptide release and, in turn, differentially modulate aversive responses mediated primarily by the two ASH sensory neurons. For example: 1) the ASI OA receptor, SER-6, and neuropeptides encoded by *nlp-6*, *-7* and *-9* are essential for the OA inhibition of aversive responses to 100% 1-octanol [6], 2) the ASI TA receptor, TYRA-3, and neuropeptides encoded by *nlp-1*, *-14* and *-18* are essential to inhibit the 5-HT stimulation of aversive responses to 30% 1-octanol [11], and 3) the ASI 5-HT receptor, SER-1, appears to stimulate the release of neuropeptides encoded by *nlp-24* that activate the Gα_o_-coupled ASI opiate receptor, NPR-17, to interfere with TYRA-3 signaling in response to 30% 1-octanol [5].

**Fig 2:**
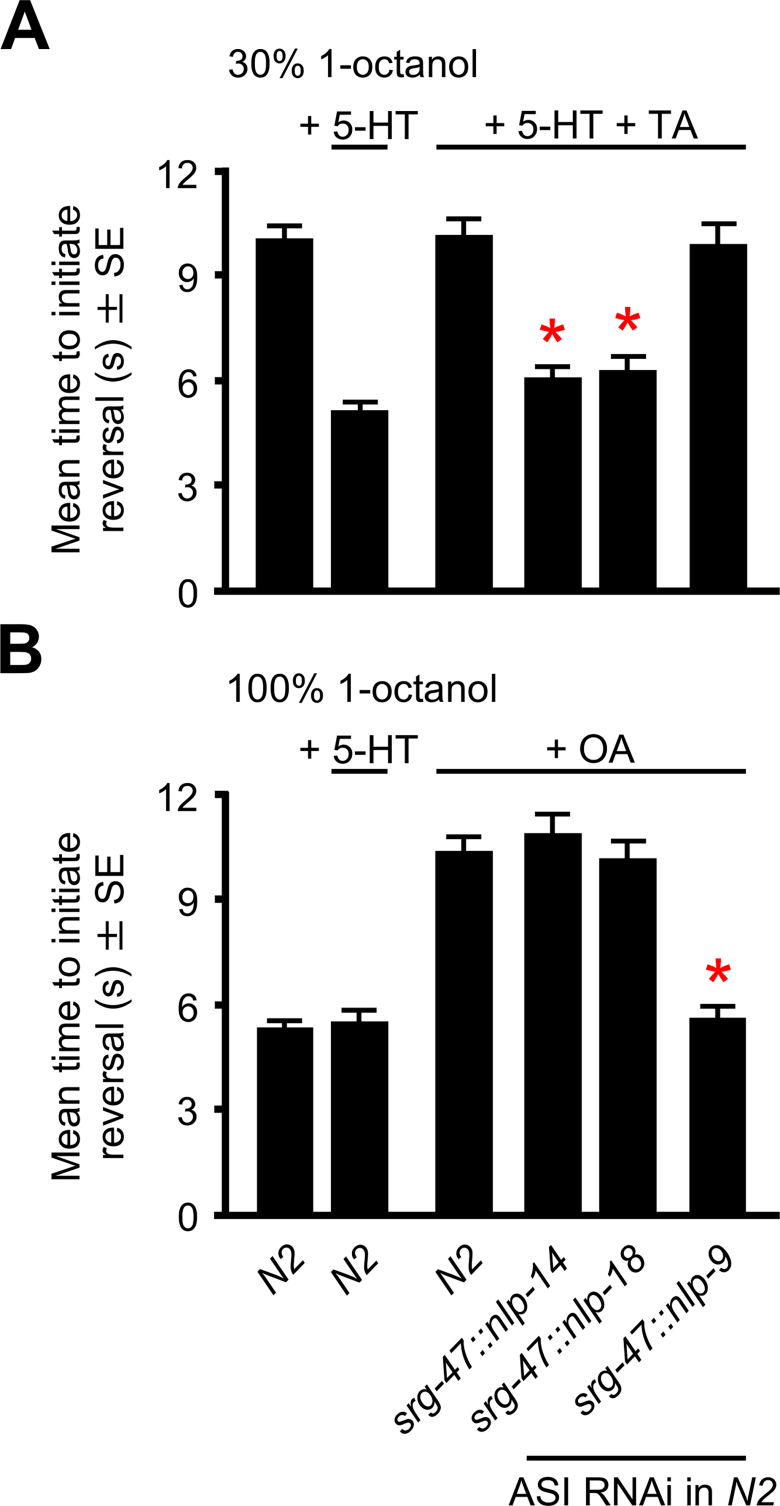
The TA- and OA-dependent inhibition of aversive responses requires different subsets of ASI neuropeptides. (A) The initiation of aversive responses to 30% (dilute) 1-octanol were assayed in the presence and absence of 5-HT and/or 5-HT + TA, as described previously [8, 11]. (B) The initiation of aversive responses to 100% 1-octanol were assayed in the presence and absence of OA, as described previously [6, 10]. The *srg-47* promoter was used to drive specific ASI RNAi expression. Monoamine concentrations were 4 mM. * Denotes significantly different from wild-type (N2) animals under the same conditions (*p* = 0.0001). Data are presented as a mean ±SE (*n*) and were analyzed by two-tailed Student’s *t* test (2A *n* = 29–34. 2B *n* = 31–36).

The two ASI sensory neurons express over 20 neuropeptide genes that encode over 60 predicted neuropeptides [20, 21]. Our previous work using primarily null mutants and ASI-specific rescue demonstrated that the serotonergic, tyraminergic and octopaminergic modulation of ASH-dependent nociception requires distinct subsets of these neuropeptide-encoding genes and suggested that monoamines might differentially modulate neuropeptide release, as outlined in Fig 1 [5, 6, 9, 11]. For example, the Gα_q_-coupled TA receptor, TYRA-3, and ASI neuropeptides encoded by *nlp-1*, *-14* and *-18* are required for the TA inhibition of 5-HT stimulation [11]. In agreement, RNAi knockdown of *nlp-14* and *-18* using a different ASI-specific promoter abolished the TA inhibition of 5-HT-stimulated aversive response (Student’s *t*-test: *srg-47*::*nlp-14 p =* 0.0001; *srg-47*::*nlp-18 p =* 0.0001; Fig 2A; [11]). It was important to use a second ASI-specific promoter to confirm previous results in order to reduce the potential for any artifacts associated with RNAi before we proceeded further. For example, even though the two promoters exhibit ASI specificity when coupled to sequence coding for GFP, it is possible that the additional sequence coding for the RNAi could alter this specificity. Similarly, the α_1_-adrenergic-like Gα_q_-coupled OA receptor, SER-6, and ASI neuropeptides encoded by *nlp-6*, *-7* and *-9* are required for the OA inhibition of the aversive response to higher 1-octanol concentrations. Again, in agreement, RNAi knockdown of *nlp-9* abolished the OA inhibition of the aversive response (Student’s *t*-test: *srg-47*::*nlp-9 p =* 0.0001; Fig 2B; [6]). Together, these data suggest that monoamine-dependent Gα_q_-coupled signaling in the ASIs differentially modulates ASI neuropeptide signaling and subsequently aversive behavior. Therefore, the present study was designed to build upon our previous findings to understand how TA and OA might alter neuropeptide signaling from the ASIs, focusing specifically on ASI neuropeptides encoded *nlp-9*, *-14* and -*18*.

### TA- and OA-dependent neuropeptides appear to co-localize at individual ASI synapses

**Fig 3:**
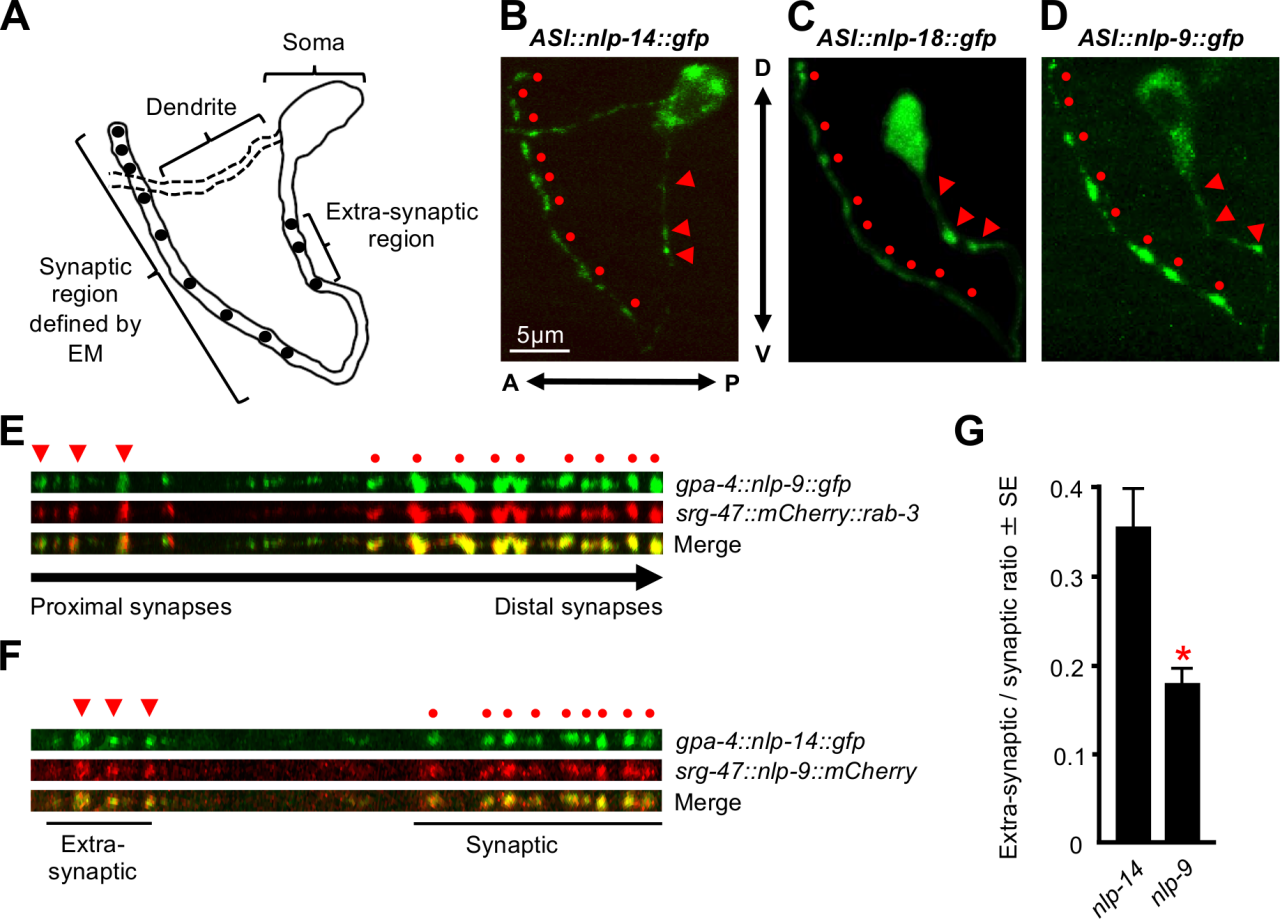
TA- and OA-dependent neuropeptides co-localize at ASI synapses. (A) Schematic representation of an ASI neuron highlighting the predicted synaptic/perisynaptic and extra-synaptic sites. (B-D) Translational neuropeptide fusions (ASI::*nlp-14*::*gfp*, ASI::*nlp-18*::*gfp* or ASI::*nlp-9*::*gfp)* expressed in their respective null backgrounds in the left ASI sensory neurons driven by the ASI-specific promoter *gpa-4*. Anterior is to the left and dorsal is up. (E) Straightened axons of *nlp-9* null animals co-expressing *gpa-4*::*nlp-9*::*gfp* and *srg-47*::*mCherry*::*rab-3* transgenes. Circles indicate the sites of predicted ASI synapses based on electron microscopy. Arrowheads denote potential additional extra-synaptic sites of release. (F) Straightened axons of an animal co-expressing *gpa-4*::*nlp-14*::*gfp* and *srg-47*::*nlp-9*::*mCherry* transgenes. (G) Extra-synaptic/synaptic ratios of ASI::*nlp-14*::*gfp* or ASI::*nlp-9*::*gfp* expressed in their respective null backgrounds. * Denotes significantly different to *nlp-14* (*p* = 0.0001). Data are presented as a mean ±SE (*n*) and were analyzed by two-tailed Student’s *t* test (*n* = 28–55).

The ASIs contain 7–9 synapses in the distal portion of the axonal process based on serial electron microscopy reconstructions and confirmed by 7–9 fluorescent puncta found after ASI expression of synaptobrevin::GFP, a synaptic marker (Fig 3A; [22, 23]). In addition, we previously demonstrated NLP-14::GFP fluorescence was observed in 7–9 discrete puncta along the distal portion of ASI axons that co-localized with the synaptic marker, RAB-3 (Fig 3B circles; [11]). In the present study, we have confirmed this observation and demonstrated that fluorescence from NLP-18:: and NLP-9::GFP was also observed in 7–9 fluorescent puncta in the distal portions of ASI axons (Fig 3C and 3D circles). As observed previously for NLP-14::GFP, NLP-9::GFP also co-localized with a synaptic marker, mCHERRY::RAB-3, within these predicted synaptic regions at least at the level of fluorescence microscopy (Fig 3E circles; [11]). However, since neuropeptide-transporting dense core vesicles (DCVs) often localize perisynaptically, the potential for a non-overlapping perisynaptic localization of the individual peptide-containing vesicles should not be ruled out [24, 25]. NLP-14::GFP and NLP-9::mCHERRY also co-localized at fluorescent puncta within predicted synaptic/perisynaptic regions of the ASIs, as anticipated, based on their co-localization with RAB-3 (Fig 3F circles; [11]). Together, these data suggest that both TA- and OA-dependent neuropeptides co-localize to each of 7–9 synaptic/perisynaptic puncta within the distal portion of right and left ASI axons. Interestingly, although all of the animals expressing ASI::peptide::GFP exhibited 7–9 fluorescent synaptic/perisynaptic puncta, the spacing of these puncta in either the right or left ASIs often varied significantly from animal to animal, making the correlation of individual fluorescent puncta with specific downstream synaptic partners problematic. Whether these differences result from preparation artifacts is unclear, but it is worth noting that most of what we know about synaptic connectivity in *C*. *elegans* is derived from very few animals and it would not be surprising if the physical relationship among ASI synapses varied from animal to animal.

Additional peptide::GFP and mCHERRY::RAB-3 fluorescence was also observed in the soma and co-localized in the axon proximal to the cell body, where no synapses have been described by electron microscopy (Fig 3B–3F, arrowheads). Whether these axonal fluorescent puncta represent trafficking vesicles, additional synapses not observed by electron microscopy or sites of extra-synaptic release remains to be determined, but the localization of at least some of these puncta appeared to be consistent in ASIs expressing different peptide::GFPs. For example, fluorescence from NLP-9::, NLP-14:: and NLP-18::GFP was consistently observed in 3–4 distinct puncta in the proximal portion of the axon near the soma (Fig 3B–3D, arrowheads). Similar ASI fluorescent puncta have also been observed by others after ASI expression of synaptobrevin::GFP, another synaptic marker [22]. Interestingly, the ratio of GFP fluorescence in synaptic and predicted extra-synaptic puncta differed significantly in ASIs from animals expressing either the TA-dependent NLP-14::GFP or the OA-dependent NLP-9::mCHERRY, suggesting that these TA- and OA-dependent neuropeptides may be trafficked in different vesicles or preferentially localized to different sites (Student’s *t*-test: *nlp-9 p =* 0.0001; Fig 3G).

### TA and OA stimulate the release of distinct subsets of ASI neuropeptides

**Fig 4:**
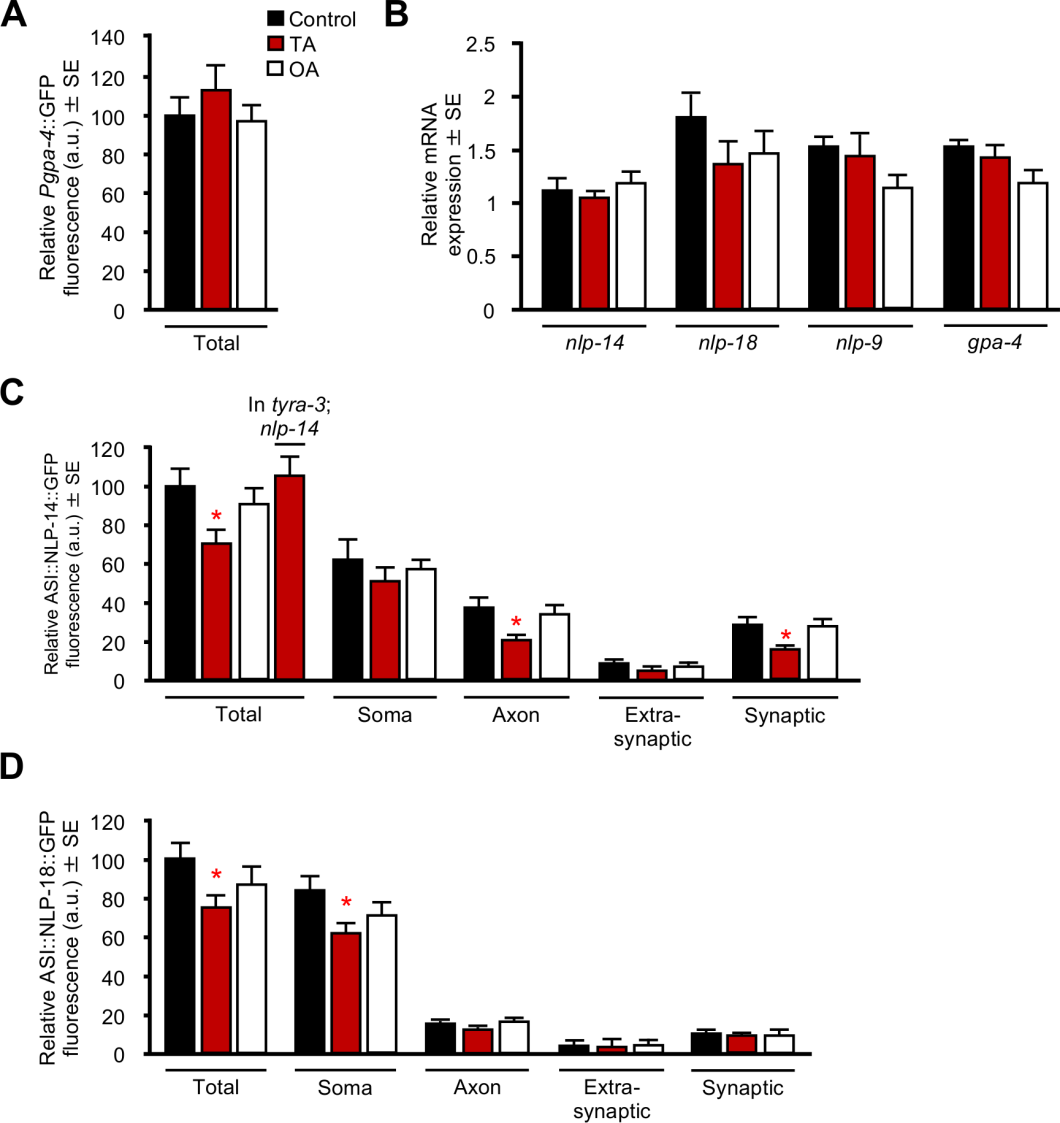
TA modulates ASI neuropeptide release. (A) Wild-type animals expressing *Pgpa-4*::*gfp* were incubated with either TA or OA and analyzed for GFP fluorescence. (B) Quantitative PCR of several ASI neuropeptide-encoding genes and *gpa-4* in wild-type animals. (C-D) Animals expressing ASI::*nlp-14*::*gfp* or ASI::*nlp-18*::*gfp* transgenes in their respective null backgrounds were incubated with either TA or OA and analyzed for GFP fluorescence. (C) ASI::*nlp-14*::*gfp* in *nlp-14* or *nlp-14;tyra-3* null animals. (D) ASI::*nlp-18*::*gfp* in *nlp-18* null animals. The *gpa-4* promoter was used to drive ASI expression. All monoamine concentrations were 10 mM. * Denotes significantly different from control (*p ≤* 0.05). Data are presented as a mean ± SE (*n*) and were analyzed by One-way ANOVA (4A *n* = 31–33. 4B *n* = 9. 4C *n* = 22–28 One-way ANOVA: Total = *p* 0.020, Dunnett *t* (2-sided) = *p* 0.037, Axon = *p* 0.006, Dunnett T3 = *p* 0.011, Synaptic = *p* 0.006, Dunnett T3 = *p* 0.012. 4D *n* = 17–45 One-way ANOVA: Total = *p* 0.045, Dunnett *t* (2-sided) = *p* 0.025, Soma = *p* 0.035, Dunnett *t* (2-sided) = *p* 0.019).

No methods have been described to measure acute neuropeptide release in *C*. *elegans*, but two indirect methods have been used previously to approximate release; treatment-dependent decreases in neuronal-specific peptide::GFP fluorescence or increases in fluorophore uptake by coelomocytes, scavenger cells located in the pseudocoelmic fluid [26–29]. Unfortunately, the coelomocyte uptake assay is not effective for all neuropeptides because uptake is dependent on the amount of neuropeptide released and the location of the neuropeptide-releasing cells [26]. Indeed, the coelomocyte uptake assay was not useful for measuring the release of ASI neuropeptides encoded by *nlp-9*, *-14 or -18* in the present study, as coelomocyte uptake was low, inconsistent and different pairs of coelomocytes sometimes yielded different results. Therefore, we focused on monoamine-dependent decreases in fluorescence from GFP-tagged neuropeptides to approximate peptide release, using a monoamine-insensitive ASI-specific promoter, *gpa-4*. Worms were transferred to a monoamine-supplemented plate for 1 hr prior to imaging for all assays, and as noted in Fig 4A, TA and OA had no effect on ASI *gpa-4*::GFP expression. In addition, TA and OA also had no significant effect on *gpa-4*, *nlp-14*, *-18* and *-9* expression as assayed by qPCR, not surprisingly given the short time course of the experiment (Fig 4B).

Our behavioral data demonstrated that neuropeptides encoded by both *nlp-14* and *nlp-18* are required for the TA-inhibition of aversive responses (Fig 2; [11]). As predicted, TA significantly decreased total NLP-14::GFP fluorescence of *nlp-14* null animals expressing ASI::NLP-14::GFP (One-way ANOVA: Total *p =* 0.020; Fig 4C). TA-dependent decreases in total ASI::NLP-14::GFP fluorescence required the TA receptor TYRA-3, as TA had no effect in *nlp-14;tyra-3* null animals lacking the key TA receptor essential for the TA inhibition of aversive responses (Fig 4C; [11]). In contrast, OA had no effect on ASI::NLP-14::GFP fluorescence (Fig 4C). Together, these data demonstrate that decreases in ASI::NLP-14::GFP fluorescence were both TA-specific and TYRA-3-dependent. Importantly, TA, but not OA, also specifically decreased axonal GFP fluorescence in predicted synaptic/perisynaptic, but not extra-synaptic regions (One-way ANOVA: Axonal *p =* 0.006; Synaptic *p =* 0.006; Fig 4C). These TA-dependent decreases in ASI::NLP-14::GFP axonal fluorescence suggest either that TA reduces NLP-14::GFP expression and/or stimulates NLP-14::GFP release, but since TA had no effect on the ASI::GFP fluorescence driven by the *gpa-4* promoter, we postulate that these decreases in peptide::GFP fluorescence were due to TA-dependent increases in synaptic/perisynaptic neuropeptide release. Indeed, neuropeptide release has been previously observed *in vitro* using *C*. *elegans* neurons. For example, Smith *et al*., observed stimulus-dependent decreases in FLP-17::VENUS fluorescence from cultured BAG neurons [30]. Similarly, Zhou *et al*., employed total internal reflection fluorescence microscopy combined with capacitance measurements to observe DCV exocytosis in cultured *C*. *elegans* neurons [31].

TA decreased total and somatic ASI::NLP-18::GFP fluorescence, but had no effect on fluorescence in ASI axonal regions (One-way ANOVA: Total *p =* 0.045; Somatic *p =* 0.035; Fig 4D). In contrast, OA had no effect on ASI::NLP-18::GFP fluorescence (Fig 4D). These observations suggest that the sites of TA-dependent ASI::NLP-18::GFP release may be somatic and different from those of NLP-14::GFP. Indeed, stimulus-dependent somatic DCV exocytosis has been observed in other systems [32, 33].

**Fig 5:**
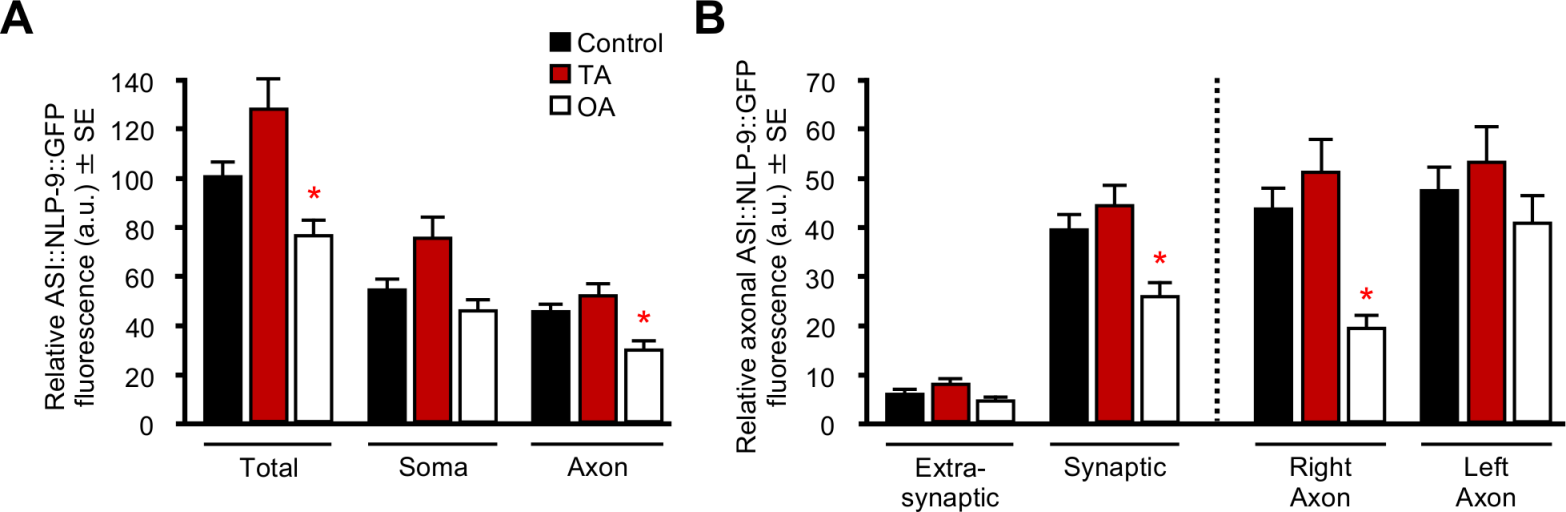
OA modulates ASI neuropeptide release. Animals expressing ASI::*nlp-9*::*gfp* transgenes in their respective null background were incubated with either TA or OA and analyzed for GFP fluorescence. (A) ASI::*nlp-9*::*gfp* in *nlp-9* null animals. (B) Axonal analysis of ASI::*nlp-9*::*gfp* fluorescence in extra-synaptic/synaptic sites and right/left ASIs. The *gpa-4* promoter was used to drive ASI expression. All monoamine concentrations were 10 mM. * Denotes significantly different from control (*p ≤* 0.05). Data are presented as a mean ± SE (*n*) and were analyzed by One-way ANOVA (5A *n* = 38–55 One-way ANOVA: Total *p =* 0.0001, Dunnett T3 = *p* 0.028, Axon *p =* 0.0001, Dunnett *t* (2-sided) = 0.004. 5B *n* = 20–55 (L = 20–28, R = 18–28), One-way ANOVA: Synaptic *p =* 0.0001, Dunnett *t* (2-sided) = *p* 0.003, ASIR *p =* 0.0001, Dunnett T3 *p* = 0.0001).

Although OA had no effect on ASI::NLP-14::GFP or ASI::NLP-18::GFP fluorescence, OA significantly reduced total and axonal ASI::NLP-9::GFP fluorescence (One-way ANOVA: Total *p =* 0.0001; Axon *p =* 0.0001; Fig 5A). Interestingly, this decrease in NLP-9::GFP axonal fluorescence was observed only in synaptic/perisynaptic puncta in the right, but not left ASI, suggesting that ASI signaling was asymmetric (One-way ANOVA: Synaptic *p =* 0.0001; ASIR *p =* 0.0001; Fig 5B). As predicted, TA had no effect on total, somatic or axonal NLP-9::GFP fluorescence. Together, these studies demonstrate TA and OA differentially modulate the release of distinct subsets of neuropeptides from different sites with the ASIs.

### ASI neuropeptide proproteins required for either TA- or OA-dependent inhibition contain different leader sequences

**Fig 6:**
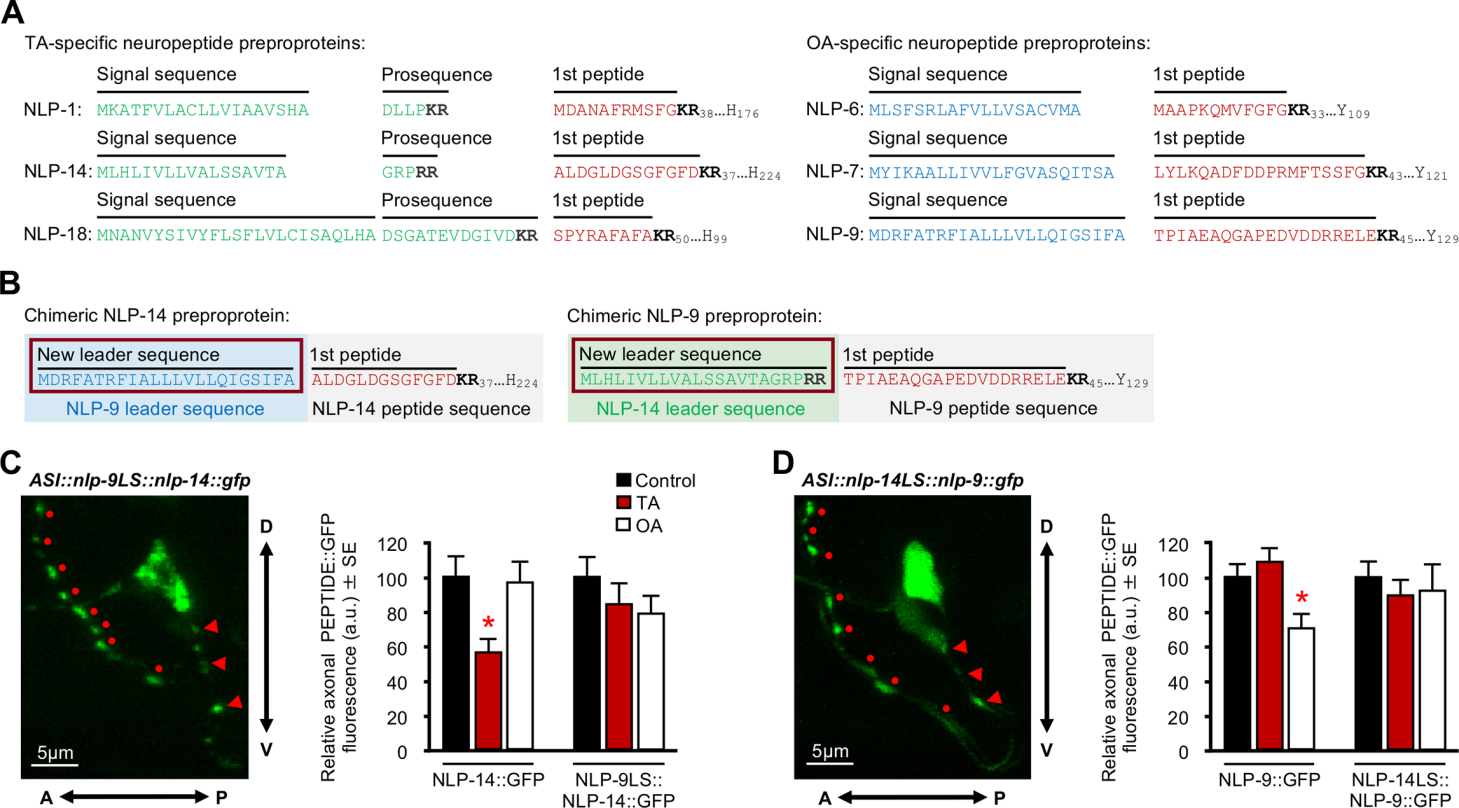
Effect of TA and OA on ASI GFP fluorescence in animals expressing NLP-14/NLP-9 chimeric neuropeptides. (A) N-terminal amino acid sequences of the TA- and OA-specific neuropeptide preproproteins. (B) Schematic representation depicting chimeric NLP-14/NLP-9 preproproteins after swapping N-terminal preprosequences. (C-D) Chimeric ASI::*neuropeptide*::*gfp* transgenes expressed in their respective null backgrounds. Anterior is to the left and dorsal is up. Circles indicate the sites of predicted ASI synapses based on electron microscopy. Arrowheads denote potential additional extra-synaptic sites of release. Chimeric fusions were incubated with either TA or OA and analyzed for GFP fluorescence. (C) Chimeric ASI::*nlp-9LS*::*nlp-14*::*gfp*. (D) Chimeric ASI::*nlp-14LS*::*nlp-9*::*gfp*. The *gpa-4* promoter was used to drive ASI expression. All monoamine concentrations were 10 mM. * Denotes significantly different from control (*p ≤* 0.05). Data are presented as a mean ± SE (*n*) and were analyzed by One-way ANOVA (6C *n* = 22–36, One-way ANOVA: NLP-14 *p* 0.012, Dunnett T3 = *p* 0.025. 6D *n* = 31–55, One-way ANOVA: NLP-9 *p* 0.003, Dunnett *t* (2-sided) = *p* 0.014).

The N-terminal sequence of neuropeptide preproproteins can be critical for efficient packaging and sorting into DCVs [34–36]. Therefore, we hypothesized that the ASI neuropeptides released by TA may localize to different DCVs than those released by OA and analyzed each of the predicted ASI neuropeptide preproproteins for clusters of charged amino acids similar to those involved in the trafficking of protachykinin. For example, charged N-terminal elements sort protachykinin into DCVs and neutralization of these charged elements dramatically reduce sorting efficiency [37]. Importantly, the identity of the N-terminal mature peptides encoded by *nlp-1*, *-14*, *-18*, *-6*, *-7* and *-9* have each been confirmed by mass spectrometry [21, 38]. Each of the three TA-dependent preproproteins (encoded by *nlp-1*, *-14* and *-18*) possessed dibasic cleavage sites upstream of the first N-terminal peptide, and after removal of the predicted signal peptide, each of the TA-dependent proproteins is predicted to contain a short, charged, N-terminal prosequence upstream of the first dibasic cleavage site (Fig 6A). In contrast, each of the three OA-dependent preproproteins (encoded by *nlp-6*, *-7* and *-9*) lack dibasic cleavage sites upstream of the first N-terminal peptide (Fig 6A). Presumably, the signal peptidase truncates these preproproteins immediately upstream of the first mature neuropeptide, leaving no charged prosequence, in contrast to proproteins encoded by *nlp-1*, *-14* and *-18*.

**Fig 7:**
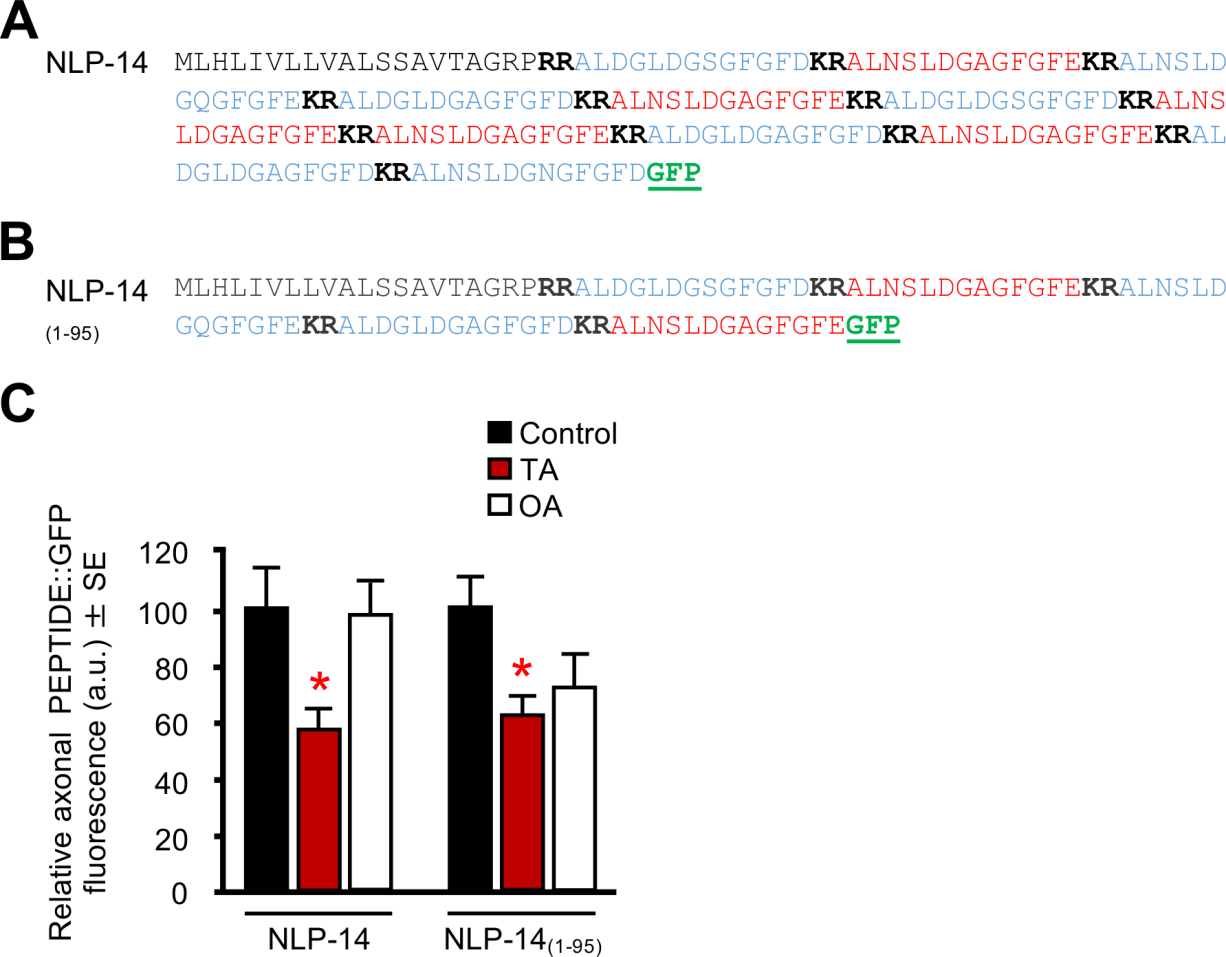
Truncation of the ASI::NLP-14 preproprotein does not alter TA and OA sensitivity. (A) Amino acid sequence for the full length NLP-14 preproprotein. (B) Amino acid sequence for the truncated NLP-14. Bold denotes a dibasic cleavage site, blue denotes putative neuropeptides, red denotes neuropeptides confirmed by either Edman degradation, Matrix Assisted Laser Desorption/Ionization-Time of Flight Mass Spectrometry, or Quadrupole-Time of Flight Mass Spectrometry, and GFP sequence is underlined green. Amino acid sequences adapted from [21]. (C) Animals expressing the truncated ASI::*nlp-14*_*(1–95)*_::*gfp* transgene in an *nlp-14* null background were incubated with either TA or OA and analyzed for GFP fluorescence. The *gpa-4* promoter was used to drive ASI expression. * Denotes significantly different from control (*p ≤* 0.05). Data are presented as a mean ± SE (*n*) and were analyzed by One-way ANOVA (7C *n* = 22–42, One-way ANOVA: Native NLP-14: *p =* 0.012, Dunnett T3 = *p* 0.025, NLP-14_(1–95)_ *p =* 0.022, Dunnett T3 = *p* 0.012).

To determine if these different preprosequences were involved in the differential action of TA and OA signaling, we constructed chimeric neuropeptide preproproteins by placing the predicted NLP-9 preprosequence upstream of the region coding for NLP-14 neuropeptides (ASI::NLP-9LS::NLP-14::GFP) and the predicted NLP-14 preprosequence upstream of the region coding for the NLP-9 neuropeptides (ASI::NLP-14LS::NLP-9::GFP) (Fig 6B). Both chimeric neuropeptides localized to the 7–9 synaptic/perisynaptic axonal puncta observed for the native GFP-tagged neuropeptides, but TA or OA did not selectively decrease axonal fluorescence from either chimera (One-way ANOVA: Native NLP-14 *p =* 0.012 Fig 6C; Native NLP-9 *p =* 0.003 Fig 6D). Together, these data suggest that the differential effects of TA and OA on neuropeptide release do not lie exclusively within the different preprosequences. If all the information required for monoamine-specific neuropeptide release was contained within the preprosequence, we predict that the chimeric peptides would be differentially released in response to the appropriate monoamine-stimulus i.e. ASI::NLP-9LS::NLP-14::GFP should be sensitive to OA, not TA, and *vice versa*. However, our data did not reflect this, suggesting additional sequence within the peptide-encoding region are required. Alternatively, the chimeric peptides may not be properly processed and/or have altered folding, with either outcome inhibiting peptide release. Further investigation is required to understand how the N-terminal sequence functions in monoamine-specific neuropeptide release, as these properties may occur at the level of DCV packaging or downstream at the site of DCV localization/exocytosis. To further assess the importance of sequence downstream of the preprosequences, we generated a truncated ASI::NLP-14::GFP protein. The *nlp-14* gene encodes seven predicted and five confirmed neuropeptides (Fig 7A; [21]). The truncated NLP-14_(1–95)_::GFP, containing the complete N-terminal sequence, three predicted neuropeptides and two confirmed neuropeptides, mimicked the full length ASI::NLP-14::GFP and exhibited decreased axonal fluorescence following incubation in TA, but not OA (One-way ANOVA: Native NLP-14: *p =* 0.012; Fig 7C; NLP-14_(1–95)_ *p =* 0.022; Fig 7C). These data suggest that specific amino acid arrangements between the leader sequence and peptide-encoding regions are important for correct packaging, processing and/or monoamine sensitivity for release, i.e. in the case of NLP-14, the first 95 amino acids confer TA-dependent release and, in contrast, disruption of this sequence, as shown by the chimeric proproteins, abolishes neuropeptide release.

## Discussion

**Fig 8:**
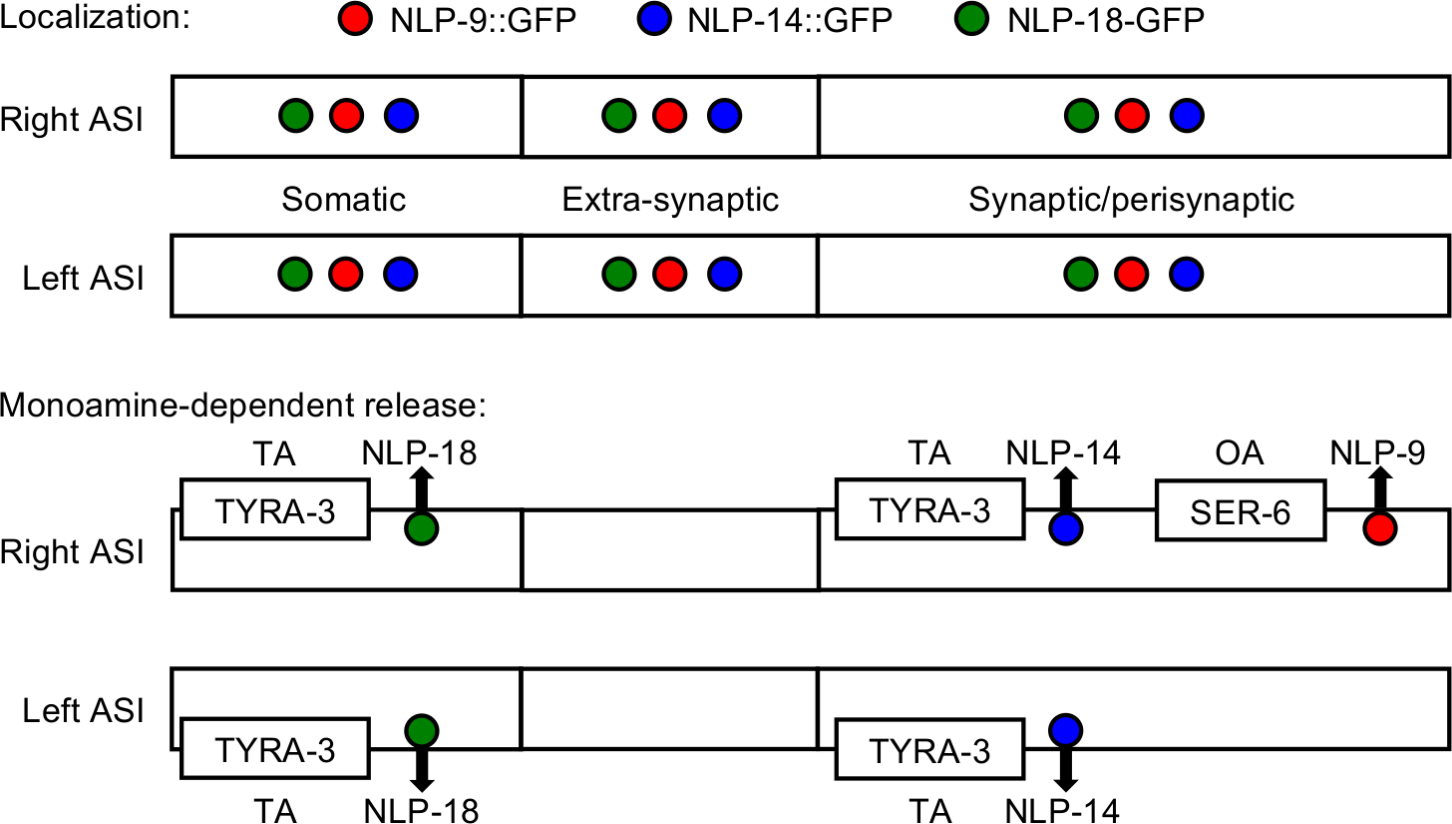
Model of monoamine-specific ASI neuropeptide release. Neuropeptides encoded by *nlp-9*, *-14* and *-18* are expressed in the soma and predicted synaptic/perisynaptic and extra-synaptic sites in both ASI neurons. TA selectively stimulates the release of NLP-18::GFP from ASI soma and NLP-14::GFP from synaptic/presynaptic regions of both the right and left ASI axons. In contrast, OA selectively stimulates the asymmetric release of NLP-9::GFP from only synaptic/perisynaptic regions in the right ASI axon.

The present study highlights the crosstalk between monoaminergic and peptidergic signaling in the modulation of *C*. *elegans* nociceptive behavior, and dissects differential monoamine-dependent neuropeptide release from a pair of sensory neurons. The monoaminergic modulation of the aversive responses to 1-octanol is dependent on differential peptidergic signaling from the two ASIs, as outlined in Fig 1 [5, 6, 11]. The present study suggests that individual monoamines selectively stimulate the release of distinct subsets of ASI neuropeptides from unique subcellular sites, with TA-dependent neuropeptides encoded by *nlp-14* and *nlp-18* released at synaptic/perisynaptic sites or the soma, respectively, and OA-dependent neuropeptides encoded by *nlp-9* released asymmetrically only from synaptic/perisynaptic sites on the right axon (Fig 8).

### Monoaminergic/Peptidergic crosstalk is extensive and modulates many key behaviors

The ASIs express Gα_q_-coupled monoamine receptors that selectively stimulate the release of different subsets of ASI neuropeptides to modulate aversive responses. These neuropeptides operate at multiple levels within the aversive circuit and are part of a large modulatory network that define behavioral state, such as nutritional status [5, 6, 11]. Monoaminergic/peptidergic interactions also modulate additional behaviors in *C*. *elegans*, including nociception, feeding, locomotion, egg-laying and mating [15, 39–42]. Monoaminergic/peptidergic signaling also interact to modulate behaviors in mammals, including fear, satiety and depression [2, 43–45]. For example, dysfunctional serotonergic and peptidergic signaling are associated with depression. Many antidepressants increase 5-HT by inhibiting 5-HT reuptake and neuropeptide Y (NPY) mimics selective 5-HT reuptake inhibitors in a rat model for depression [2, 46, 47]. In contrast, depression-like behaviors are attenuated in NPY_1_, _-2_ or _-4_ knockout mice, highlighting the complexity of the interactions [48–50]. Collectively, these studies demonstrate that monoamines and neuropeptides interact to modulate many of the same behaviors. In fact, wide-spread, complex crosstalk between these different signaling systems modulates the activity of individual neurons, entire neuronal circuits and, as we present here, interacting directly, with monoamines differentially regulating neuropeptide release from distinct sites within a single pair of neurons.

### Both TA- and OA-dependent ASI neuropeptides co-localize with synaptic/perisynaptic markers in the ASIs

Both TA- and OA-dependent neuropeptides co-localized with synaptic markers in the ASIs, although whether this localization is synaptic or perisynaptic is unclear. Indeed, neuropeptide-containing DCVs often localize perisynaptically [51]. For example, DCVs do not cluster at active zones in *C*. *elegans* cholinergic motor neurons, *Aplysia* motor neurons or the mammalian dorsal root ganglion [24, 25, 52, 53]. Discrete neuropeptide-containing DCV populations have also been identified within the same cell. AtT20 cells expressing prothyrotropin-releasing hormone exhibit distinct DCV populations containing either N- or C-terminal peptides cleaved from the original prothyrotropin-releasing hormone [54]. In the present study, neuropeptide-positive puncta were also observed along ASI axons where synapses were not identified. Whether these extra-synaptic neuronal puncta are associated with DCV trafficking or novel sites of release requires further investigation; however, they do not appear to be sites of monoamine-dependent release.

### TA and OA differentially modulate neuropeptide release from the ASIs

The monoamine selectivity of ASI neuropeptide release is dependent on the expression of different Gα_q_-coupled monoamine receptors. Since Gα_q_ signaling increases IP_3_-mediated calcium (Ca^2+^) release, we hypothesized that TA and OA stimulate local ASI Gα_q_ signaling and increase Ca^2+^, stimulating the release of DCVs from distinct pools [55, 56]. Together these observations suggest that ASI GPCRs are differentially localized and ASI Ca^2+^ signaling compartmentalized. However, compartmentalized ASI GPCR signaling or Ca^2+^ signaling have yet to be characterized directly. In contrast, localized Ca^2+^ release has been observed in a variety of other systems, with G-protein-coupled receptors often in proximity to local Ca^2+^ stores [57]. In *C*. *elegans*, a muscarinic-like G-protein-coupled receptor modulates local Ca^2+^ dynamics in the RIA interneurons to coordinate locomotion [58]. In *Drosophila*, OA stimulates neuropeptide release by activating both a cAMP-dependent protein kinase and increasing Ca^2+^ release from internal stores [59]. In mammalian retinal starburst amacrine cells, a localized Ca^2+^ signal is also compartmentalized by mGluR2 signaling that restricts propagation [60, 61]. Amacrine cells are similar to many *C*. *elegans* neurons in that they share both synaptic inputs and outputs along their neuronal processes. Similarly, many GPCRs are localized subcellularly. For example, the cannabinoid receptor, CB1R, and the somatostatin receptor, SSTR2, differentially localize to axonal and somatodendritic regions in mammalian cells, respectively [62]. Selectively modulating local Ca^2+^ dynamics could expand the signaling capacity of individual neurons, an approach potentially significant in an organism like *C*. *elegans* with only 302 neurons.

Monoamines have been shown to modulate neuropeptide release in other *C*. *elegans* behaviors/processes. For example, 5-HT stimulates the release of *flp-7* encoded peptides to decrease body fat and peptides encoded by *daf-7* and *-28* to facilitate exit from dauer [63–65]. Similarly, dopamine stimulates the release of *nlp-12* encoded peptides to modulate local search behavior and inhibits the release of *flp-8* encoded neuropeptides to modify copper aversion [29, 66]. In *Drosophila*, OA stimulates the release of GFP-tagged atrial natriuretic factor [59]. In mammals, multiple 5-HT receptors also modulate neuropeptide release associated with feeding. 5-HT stimulates the release of α-melanocyte stimulating hormone to stimulate feeding and inhibits the release of the orexigenic peptides, NPY and agouti-related protein to inhibit feeding [45, 67–69].

Conversely, neuropeptides directly modulate monoamine release. For example, *nlp-7*, *flp-10*, *-11* and *-17* encoded neuropeptides inhibit 5-HT release to inhibit egg-laying and DAF-7 inhibits TA/OA release to promote satiety and quiescence [13, 41, 70, 71]. In mammals, neuropeptide S inhibits the release of 5-HT and noradrenaline from mouse amygdala and prefrontal cortex synaptosomes, but stimulates the release of dopamine when administered intracerebroventricularly, highlighting the complex interaction between the two signaling systems [72–74]. Similarly, peptide YY (3–36) inhibits the release of dopamine and norepinephrine, but not 5-HT from isolated hypothalamic synaptosomes, suggesting that neuropeptides can confer specificity to monoamine release [75]. Collectively, these studies demonstrate that monoaminergic and peptidergic signaling often modulate the same behaviors, but also interact directly to modulate the others release.

### OA asymmetrically stimulates neuropeptide release from the ASIs

OA asymmetrically modulates the release of *nlp-9* encoded neuropeptides, with OA only stimulating release from the right ASIs. Indeed, the right and left ASIs exhibit different synaptic connectivities [23]. However, some/most neuropeptides and, indeed, most monoamines act at receptors distantly localized from their sites of release, bringing into question the potential significance of our observed spatial patterns and asymmetric peptide release, if this peptidergic signaling is extra-synaptic. Little is known about the localization of neuropeptides with their cognate receptors in *C*. *elegans*. Certainly, in *C*. *elegans* some/most neuropeptides and, indeed, most monoamines appear to signal extra-synaptically and act at receptors distantly localized from their sites of release, based on the localization of monoamine synthesizing neurons and monoamine receptors. Indeed, most monoaminergic neurons do not synapse directly onto neurons expressing most monoamine receptors [76–78]. For example, none of the monoamine-synthesizing neurons synapse directly on the ASIs and only the serotonergic ADFs synapse on the ASHs, even though these two pairs of sensory neurons express a number of 5-HT, TA, OA and dopamine receptors (available from: http://wormweb.org/neuralnet#c=ASI&m=1). However, 1) some peptide receptors may be synaptic and focus more local, extra-synaptic, monoaminergic signaling or, more likely, 2) given the tight packaging of neurons in the *C*. *elegans* nerve ring, the site of neuropeptide or monoamine release may be relevant, even for extra-synaptically localized peptide/monoamine receptors. Indeed, peptide receptors have been identified in close proximity to sites of peptide release in other systems. For example, the tachykinin-related peptide 1 and its receptor, LTKR, arborize in close proximity in the cockroach brain [79]. In *Drosophila*, immunoreactivity-labeling of tachykinins was distinct and closely resembled that of their receptor, DTKR, suggesting a local site of activation [80]. In addition, the selective localization of neuropeptidases could further modify peptidergic signaling, especially if the gene encodes 1) a mix of receptor agonists that activate different receptors or 2) agonists/antagonists that have the potential to be differentially degraded, as we have suggested previously for *C*. *elegans* opiate signaling [5]. For example, nematode neprilysins are differentially localized and modulate the degradation of number of regulatory peptides [81, 82]. No studies to date have demonstrated compartmentalization in the ASIs, but data from the RIAs and the functional asymmetry of other sensory neurons increases the plausibility of a similar situation for the ASIs. Indeed, asymmetry in both connectivity and function has been observed in other *C*. *elegans* neuron pairs. For example, the differential expression of guanylyl cyclases contributes to functional asymmetry in the two ASE sensory neurons [83–86]. Similarly, the two functionally distinct AWC sensory neurons asymmetrically express *str-2*, a putative olfactory receptor, specifying *str-2*-expressing cells as AWC^ON^ and non-*str-2*-expressing cells as AWC^OFF^ [87–89]. Collectively, these studies highlight the ability of individual pairs of neurons to differentially perceive and process sensory information.

### The TA- and OA-dependent ASI neuropeptides contain different predicted preprosequences

Neuropeptides are cleaved from preproproteins that generally encode multiple peptides, with packaging and trafficking information often located within the N-terminus of the preproprotein [38, 90, 91]. For example, efficient DCV packaging was observed when the first 26 N-terminal amino acids from proopiomelanocortin were fused to a reporter [92]. Indeed, the preprosequences predicted for the TA- and OA-dependent ASI neuropeptide-encoding genes differed significantly, with the TA-dependent preprosequences containing a predicted charged leader upstream of the first peptide and the OA-dependent preprosequences cleaved immediately upstream of the first peptide. However, these different preprosequences were not sufficient to confer monoamine-specificity, as chimeric NLP-9 and NLP-14 preproproteins with swapped preprosequences lost all monoamine-specificity. In contrast, the monoamine-specificity of the NLP-14 preproprotein was maintained after substantial C-terminal truncation. Similarly, proneuropeptide Y truncated at the C-termini was efficiently localized to DCVs and secreted [93]. Collectively, our data suggest that N-terminal signal sequence is not sufficient to confer monoamine-selectivity to neuropeptide release and that additional N-terminal peptide-encoding sequence is required.

In summary, the present study highlights the complexity of monoaminergic/peptidergic interactions within a single neuron pair and demonstrates that the adrenergic-like ligands, TA and OA stimulate the release of distinct subsets of neuropeptides from the ASIs to modulate aversive behavior. In addition, the sites associated with the release of neuropeptides encoded by *nlp-14*, *-18* and *-9* within the ASIs also appear to be distinct, with TA-dependent neuropeptides encoded by *nlp-14* and *nlp-18* released at synaptic/perisynaptic sites or the ASI soma, respectively, and OA-dependent neuropeptides encoded by *nlp-9* released asymmetrically only from synaptic/perisynaptic sites on the right axon.

## Materials & methods

### Nematode strains

General culture and handling of strains were maintained as previously outlined [94]. The following alleles were used: wild-type N2 *(Bristol strain)*, *nlp-9 (tm3572)*, *nlp-14 (tm1880)*, *nlp-18 (ok1557)* and *tyra-3 (ok325)*. All strains were obtained from the *Caenorhabditis* Genetics Center (University of Minnesota, Minneapolis, MN) except *nlp-9 (tm3572)* and *nlp-14 (tm1880)* which was received from the National Bio-Resources Project (Tokyo Women’s Medical University, Tokyo, Japan). Animals containing double mutations were generated using standard genetic techniques and confirmed by PCR. All mutants were backcrossed with wild-type N2 strain at least four times.

### Construction of *C*. *elegans* transgenes

The *gpa-4*::*nlp-9*::*gfp*, *gpa-4*::*nlp-14*::*gfp* and *gpa-4*::*nlp-18*::*gfp* transgenes were generated by 3-piece PCR fusion with sequence coding for GFP fused in frame to the C–terminal peptide and contained the 2.6 kb *gpa-4* promoter, cDNA of the respective neuropeptide-encoding gene and *gfp*::*unc-54 3’* UTR. Each transgene was co-injected with the selectable markers *unc-122*::*rfp*, *rol-6 (su1106)* or *f25b3*.*3*::*gfp* (to 50 ng). The *srg-47*::*nlp-9*::*mCherry* included the 750 bp *srg-47* promoter, *nlp-9* cDNA and *mCherry*::*let-858 3’* UTR; the *srg-47*::*mCherry*::*rab-3* included the 750 bp *srg-47* promoter, *mCherry*, full-length *rab-3* cDNA and *unc-54 3’* UTR. The *gpa-4*::*nlp-14[leader sequence]*::*nlp-9*::*gfp* included the 2.6 kb *gpa-4* promoter, *nlp-14* leader sequence (the first 66 bp), full-length *nlp-9* cDNA (minus the first 69 bp) and *gfp*::*unc-54 3’* UTR; the *gpa-4*::*nlp-9[leader sequence]*::*nlp-14*::*gfp* included the 2.6 kb *gpa-4* promoter, *nlp-9* leader sequence (the first 69 bp), full-length *nlp-14* cDNA (minus the first 66 bp) and *gfp*::*unc-54 3’* UTR; the truncated *gpa-4*::*nlp-14*_*(1–95)*_::*gfp* included the 2.6 kb *gpa-4* promoter, the first 285bp *nlp-14* cDNA and *gfp*::*unc-54 3’* UTR and the transcriptional *Pgpa-4*::*gfp* included 2.6 kb *gpa-4* promoter and *gfp*::*unc-54 3’* UTR. All ASI-specific RNAi transgenes were generated by PCR fusion using the 750 bp *srg-47* promoter, as described previously [11, 95] and co-injected with *f25b3*.*3*::*gfp* (to 100 ng). Carrier DNA was injected into the gonads of *C*. *elegans* wild-type or mutant backgrounds by standard techniques [96]. All PCR fusions were performed as described previously and confirmed by restriction digest [97].

### Behavioral assays

Octanol avoidance assays were performed as described previously [11, 98]. Briefly, L4 animals were picked 24 hrs prior to assay and kept at 16°C on standard nematode growth medium (NGM) seeded with *Escherichia coli* strain OP50. NGM plates were prepared daily by adding 5-HT (4 mM) and/or TA/OA (4 mM) to liquid media before pouring. A hair (Loew-Cornell 9000 Kolinsky 8 paintbrush) was fused to a P200 pipette tip and dipped in either dilute (30% v/v in 100% ethanol) or 100% 1-octanol and placed in front of an animal exhibiting forward locomotion. The time taken to initiate backward locomotion was scored for each animal and averaged. Assays were terminated after 20 sec as wild-type animals are known to spontaneously reverse ~20 sec [98]. In all assays, animals were transferred to intermediate (non-seeded NGM) plates and left for 30 sec, then transferred to assay plates and left for 30 min before being scored for 1-octanol avoidance. In all assays, 27–38 worms were examined for each strain and condition. All strains were blinded and assays repeated in triplicate. Statistical analysis was performed using individual responses ± SE and Student’s *t* test.

### Confocal microscopy

ASI::*neuropeptide*::*gfp/mCherry* transgenes were constructed by PCR fusion, as described above. To co-localize NLP-9 and RAB-3, two different ASI promoters were used; *gpa-4*::*nlp-9*::*gfp* and *srg-47*::*mCherry*::*rab-3* and co-injected into *nlp-9* null animals. A similar approach was used to co-localize NLP-9 and NLP-14 using *srg-47*::*nlp-9*::*mCherry* and *gpa-4*::*nlp-14*::*gfp*. Animals were immobilized in 20 mM sodium azide, mounted on 3% agarose pads and then imaged for GFP and mCHERRY using sequential scanning. All microscopy was performed on an Olympus IX81 inverted confocal microscope.

### Neuropeptide fluorescence assay

For all neuropeptide fluorescence assays, transgenic L4 animals expressing either: P*gpa-4*::*gfp*, *gpa-4*::*nlp-9*::*gfp*, *gpa-4*::*nlp-14*::*gfp*, *gpa-4*::*nlp-18*::*gfp*, *gpa-4*::*nlp-14[leader sequence]*::*nlp-9*::*gfp*, *gpa-4*::*nlp-9[leader sequence]*::*nlp-14*::*gfp* or *gpa-4*::*nlp-14*_*(1–95)*_::*gfp* were picked 24 hrs prior to assaying and kept at 16°C. NGM plates containing either TA or OA (10 mM) were prepared daily. Animals were incubated on NGM plates seeded with *E*. *coli* strain OP50 or transferred to assay plates for 1 hr prior to imaging. Images (0.4 μm Z-stacks) were captured using Olympus Fluoview FV1000 software and fluorescence was quantified using Volocity Software Version 4.0.0 Build 314 (Improvision Ltd). To quantify neuropeptide fluorescence, both the right and left ASIs were tightly cropped and divided into the soma and axon. All images were then blinded and fluorescence was filtered by intensity. The first 10 blinded images were used to generate an average threshold separating signal from noise, which was applied to all images within the experiment. Object intensities were summed for each image and objects < 3 voxels were regarded as non-specific background and excluded. The summed fluorescence of each treatment was normalized to the summed fluorescence of untreated (control) animals. All imaging was performed on an Olympus IX81 inverted confocal microscope and animals were immobilized in 20 mM sodium azide on 3% agarose pads. Statistical analysis was based on individual responses to treatment with *n* values given where appropriate. At least two plates per condition were used in all experiments.

### RNA isolation and cDNA synthesis

RNA was isolated as outlined previously [99] with minor revisions. Briefly, wild-type animals were synchronized using alkaline hypochlorite and arrested at L1 before being moved to fresh NGM plates seeded with *E*. *coli* strain OP50 and maintained at 16°C. Young adults were then washed in M9 and transferred to either fresh NGM plates seeded with *E*. *coli* strain OP50 (untreated population), fresh non-seeded NGM plates (control population), fresh OA (10 mM) or fresh TA (10 mM) NGM plates for 1 hr. Following incubation, animals were then washed in M9 and suspended overnight at -80°C in Trizol^™^ (Invitrogen^™^). Animals were frozen and lysed via freeze-thaw cracking in liquid nitrogen. RNA was isolated using standard Trizol^™^ techniques and assessed for quality/quantity on an agarose gel and using Nanodrop^™^ ND-1000 Spectrophotometer. cDNA synthesis was performed using the SuperScript^™^ III First-Strand Synthesis system (ThermoFisher Scientific).

### Quantitative PCR

qPCR studies were conducted using SsoFast^™^ EvaGreen® Supermix (Bio-Rad) and Bio-Rad CFX96^™^ Real-Time PCR System. qPCR reactions were set up as per manufacturer’s instructions. cDNA pools from either untreated, control, OA (10 mM) or TA (10 mM) populations were diluted to 50 ng/μl and 2 μl added for reaction set up. At least three separate pools of cDNA were analyzed. Cycling conditions were used as per the manufacturer’s instructions. All results were normalized to the reference gene *act-1* and relative expression determined using the Livak ΔCt method [100]. Data are presented as individual responses ± SE and analyzed using One-way ANOVA.

### Bioinformatic analysis

Predicted signal sequence cleavage sites were obtained using SignalP V4.1 (http://www.cbs.dtu.dk/services/SignalP/) and confirmed using Signal-BLAST (sigpep.services.came.sbg.ac.at/signalblast.html). Discrepancies between algorithms were resolved by selecting the most likely cleavage site based on the Edman degradation/mass spectrometry data detailing previously identified *C*. *elegans* neuropeptides [21].

### Statistics

All statistics were performed using SPSS software v24 (IBM). All data was initially assessed with either a Student’s *t*-test or a One-way ANOVA and when appropriate, post-hoc analysis was carried out using a Dunnett or Dunnett T3 test.

## Supporting information

S1 File Data supporting the underlying findings described within the manuscript. (XLSX)

## Abbreviations

5-HT: Serotonin
Ca^2+^: Calcium
DCV: Dense Core Vesicle
NGM: Nematode Growth Medium
NPY: Neuropeptide Y
OA: Octopamine
TA: Tyramine
